# Assessing the Health Needs of Chinese Older Adults: Findings from a Community-Based Participatory Research Study in Chicago's Chinatown

**DOI:** 10.4061/2010/124246

**Published:** 2011-01-03

**Authors:** XinQi Dong, E-Shien Chang, Esther Wong, Bernarda Wong, Kimberly A. Skarupski, Melissa A. Simon

**Affiliations:** ^1^Department of Internal Medicine, Rush University Medical Center, 1645 West Jackson Boulevard, Suite 675, Chicago, IL 60612, USA; ^2^Chinese American Service League, Chicago, IL 60616, USA; ^3^Department of Obstetrics/Gynecology, Feinberg School of Medicine, Northwestern University, Chicago, IL 60611, USA

## Abstract

The objective of this study is to examine the cultural views of healthy aging, knowledge and barriers to services, and perception of health sciences research among community-dwelling Chinese older adults in Chicago's Chinatown. This qualitative study is guided by the Precede-Proceed conceptual model with community-based participatory research design. Data analysis is based on eight focus group interviews with Chinese older (age 60+) adults (*n* = 78). We used a grounded theory framework to systematically guide the thematic structure of our data. Findings show participants described cultural conception of health in terms of physical function, psychological well-being, social support, and cognitive function. The availability, affordability, and cultural barriers towards health care services were major negative enabling factors that inhibit participants from fulfilling health needs. Perception and knowledge of health sciences research were also discussed. This study has implications for the delivery of culturally appropriate health care services to the Chinese aging population.

## 1. Introduction

Chinese Americans, the largest Chinese population outside Asia, are aging at a rapid rate. As the oldest and largest Asian American community, Chinese population currently numbers 3.6 million [1]. Compared to other Asian-American groups, Chinese community is older and has a higher median age. At the same time, over half (54.2%) of the Chinese community is composed of the less acculturated first generation [1]. 

The graying of Chinese community in the US warrants the attention of health professionals and policy makers to provide culturally sensitive healthy aging initiatives. Prior studies suggest that there are significant health disparities among Chinese persons including chronic diseases, cancer, and psychological distress after having relocated and losing contact from their native support network [2–7]. The stress of aging compounded with migration distress may make this group of immigrants even more vulnerable [8]. 

In addition, health system barriers may prevent Chinese population from getting the needed health care. Study suggests that Chinese persons are least likely to believe that their backgrounds and values are understood by US health care providers [9]. While eighty-seven percent of Chinese older adults in the US are foreign born, their conceptualization of health and health-seeking behaviors is likely to be heavily informed by traditional Chinese thinking [10].

For thousand of years, Chinese are influenced by the teachings of Confucianism and Daoism which greatly shape the way in which health, illnesses, treatment, and stress-coping tendency are conceptualized and practiced [11]. For instance, providing care to older adults is a highly regarded cultural belief and continue to play an important factor in the life of older adults and their families in Chinese culture [12]. However, as more and more Chinese families migrate from traditional Chinese collective culture to Western society's emphasis on individualism, traditional values such as respect for older adults, familial harmony and filial care may undergo constant modification and transformation. Some research suggests that US Chinese older adults' expectation of filial care has been shifted to dependence on friends and neighbors [13]. Others suggest that although the decrease of power and resources put Chinese older adults in an unfavorable position, the modified expectation of filial care may help prevent intergenerational conflicts [14]. And still others suggest that given different degrees of adherence, traditional values continue to operate in Chinese immigrant families [15]. Despite the significance of tradition, contemporary social, cultural, and familial changes they have made it increasingly difficult for older Chinese to receive desired care. They may be forced to let go of their cultural beliefs and hence are more prone to suffer from potential family conflicts in the form of migratory and psychological distress [16–19]. It is imperative to address health care system barriers with the goal to provide culturally sensitive services that effectively take into account the Chinese culture, values, health experience, and perceived health needs [20].

While there is an urgent need to investigate the needs and health priorities of Chinese older adults, research with Chinese community have been challenging. First, health data on Chinese Americans remains scarce. Most previous research compiled data from the Asian-American population as a whole despite vast subgroup heterogeneity. Health disparities among Asian subgroups are often overlooked [7]. Second, the US Chinese population is a diverse community in terms of its various languages, ethnic backgrounds, residential patterns, socioeconomic status, generations and immigration trajectories, and degrees of acculturation. The vast diversity of Chinese overseas community inherently affects their patterns of health-seeking behavior [21]. Therefore, the subpopulation differences necessitate research designs with culturally sensitive and appropriate means [22]. Third, research has shown that recruiting and retaining older research participants can be difficult, which is likely exacerbated by distrust of the government [23, 24].

As a result, our current knowledge remains limited regarding the health needs of Chinese older adults. In our community of Chicago's Chinatown, its rapid growth and high proportion of older adults particularly warrant attention [25]. Based on the latest US census, there are approximately 20,000 Chinese living in Chicago's greater Chinatown community area. This community area roughly represents 6% of the total Asian-American and Pacific-Islander population in Chicago and is the second-largest grouping of Asian-American population in any community area in Chicago [26]. However, the Chinatown community has not been fully engaged in research due, in part, to the anti-Chinese sentiment from the past that created historical distrust toward government and federally sponsored research projects [27, 28]. Barriers including language, scientific literacy, and access to care, have further excluded Chinese community to benefit from the advancement of health sciences research [29]. 

Considering the existing health problems and lack of evidence-based research among Chinese older adults, we conducted a qualitative health needs assessment among Chinese older adults. Health needs assessment is a promising instrument to systematically identify inequalities in health and access to services among minorities. Health needs assessments are especially insightful in determining the health priorities by utilizing epidemiological and qualitative methods. In particular, it helps the community members to understand health problems within their community and identify culturally appropriate strategies for health programs [30, 31].

We used the Precede-Proceed framework as a conceptual model for the needs assessment. This multidisciplinary model has proven valuable in launching community health promotion at both local and national levels [32]. Designed to identify community needs and plan educational interventions accordingly, this model suggests that local health problems can be improved by changing individuals' predisposing, enabling, and reinforcing factors. Predisposing factors include community's values, beliefs, and perceptions that affect motivation for change. Accessibility to health services and promotions are regarded as enabling factors. The consequences of one's health behavior that determine whether the action is supported positively or negatively are considered as reinforcing factor. For instance, cultural views of healthy aging may be predisposing factor to shaping one's health needs, and potential barriers to health care services may be negative enabling factors of gathering health information. The predisposing, enabling, and reinforcing factors influencing the health needs of our study population were identified through focus group interviews. The overall objectives of this project are to (1) assess perceived health issues in the community (2) gain understanding of service-utilization patterns and (3) examine perception and knowledge of health sciences research.

## 2. Materials and Methods

### 2.1. Community-Based Participatory Research Approach

Community-based participatory approach (CBPR) has been described as an important framework to understand the health needs of minority population who are underserved, and whose unique health issues are closely linked to cultural diversity [33–35]. Through community participation, the relevance of research outcome is greatly enhanced [32, 36, 37].

In this National Institutes of Health-funded partnering in research project, we practiced CBPR approach that allowed us to fully engage Chinese community throughout the process of relationship building, mutual learning, and data sharing, in which the efforts contributed to the validity of the findings interpretation and relevance of community health needs. The academic-community collaboration was initiated in 2008 by a gerontologist at Rush University Medical Center to partner with Chinese American Service League (CASL), the oldest and biggest social service agency serving Chinese Americans in the Midwest. A community advisory board (CAB) was established with the goal to involve Chinatown members who have a vested interest and understanding of the community. Enlisted from stakeholders and leaders through civic, health, social, and advocacy groups, community centers, as well as community physician and residents, and board members, CAB members then identified a list of needs assessment topics most relevant to Chinese community's concerns that were further incorporated into focus group topics. In the later stage of this project, CAB members also worked closely with investigators to review findings and reexamine study instrument to ensure cultural sensitivity and appropriateness. Overall, conducting CBPR approach allows researchers to gain knowledge and cultural awareness of community health concerns and the ability to develop appropriate research instruments. This synergetic collaboration also increases community's understanding toward research mechanism that lays the foundation of sustainable partnership.

### 2.2. Study Design and Procedure

Focus group methodology is an important qualitative research technique. Whereas questions about health, illnesses, care, and interventions are highly culturally mediated, focus group design helps to uncover the unique cultural beliefs, values, and motivations affecting one's health behavior and well being. The results often offer valuable insights on the health of an understudied population [38]. In addition, this technique is well-suited to explore health problems in a community which is relatively unknown, such as Chinese aging population [39].

Our focus group recruitment process benefited from the collaborative relationship established between academic and the community. We approached participants after their attendance in CASL-sponsored cultural activities such as calligraphy and Taichi classes according to the following eligibility criteria: (1) aged sixty years or older, (2) self-identified as Chinese, and (3) reside in Chicago. Of the 80 participants approached, 78 Chinese older adults aged sixty and older gave consent to the study. 

Prior to focus group interviews, study participants gave written consents for audio recording. All materials were prepared in simplified Chinese, traditional Chinese, and English. In order to ensure cultural sensitivity of the study, participants were then divided into eight focus groups according to their dialect of preference [40]. In total, six focus groups were conducted in Cantonese, and two in Mandarin. This preference reflects the linguistic diversity among Chinese-American community [41, 42]. 

Community stakeholders were invested in our collaborations and were instrumental in recruiting, retaining, and collecting data for the needs assessment. Focus group interviews were guided by trained facilitators who are affiliated with CASL. The involvement of facilitators with the community is crucial to gaining trust and eliciting active participation based on the rationale that Asian participants are more likely to reveal their opinions if they know the interviewers personally [40]. Participants' perception of health needs, access and barriers to health care services, and knowledge of health sciences research were explored by the following questions.

1. What does “healthy aging” mean to you?
2. What do you think are some of the biggest health problems in our community?
3. How would you describe a happy older adult in the community? What makes older adults in our community happy?
4. How would you describe an unhappy older adult in the community? What makes them upset or lonely?
5. What are your good/bad experiences in health care in the United States?
6. How would you describe an ideal health care service for Chinese older adults?
7. What do you think are the potential benefits/ problems/impact of health sciences research in our community?
8. What advice can you give us about bringing research into our community?

The length of discussions was determined by the level of interaction among participants. Facilitators proceeded with topics when responses were exhausted. Our interviews were organized in CASL facilities, a community site where participants felt most comfortable. All procedures were approved by the Rush University Medical Center Institutional Review Board.

### 2.3. Qualitative Data Analysis

Grounded theory was used to guide the qualitative data analysis in this study. It provided a general framework to develop themes and theories from the collected data [18, 43]. All focus groups were preceded in either Cantonese or Mandarin. For analysis purpose, a bilingual research assistant first transcribed audio recordings into Chinese transcripts (different dialects used the same Chinese characters), and then translated the transcripts into English. Another assistant subsequently back translated the English transcript into Chinese. Transcribed texts were further evaluated by a bilingual principal investigator to ensure that the correct meaning was evident.

The English transcripts, imported into NVivo software (NVivo, version 8) for analysis, were scrutinized for discussion on health needs. Various barriers to health care services mentioned by the participants were identified as well. Two independent coders followed grounded theory to analyze data iteratively. Each coder first independently labeled the texts with key words and phrases. The key words were coded and analyzed for emerging categories according to the Precede-Proceed framework in terms of cultural conception of health, knowledge and barriers of health services, and perception toward health sciences research. Two coders then compared and discussed their sets of categories collectively to evolve dominant themes. The categorization of each response was not finalized until two coders reached consensus. Each category was reviewed and a short summary was written for each category. Quotes from the English transcripts that captured participants' opinions and sentiments were incorporated to support each theme [18, 43, 44].

## 3. Results and Discussion

### 3.1. Participants' Characteristics

A total of 78 Chinese older adults participated in eight focus groups; 53% were female. The mean age was 74.8 years old. Participants' average length of residence in the US was 20.6 years, and the majority emigrated from Mainland China with a small portion from Hong Kong and Taiwan. 

### 3.2. Community's Cultural Views of Health (Predisposing Factors)

We explored the knowledge of and beliefs about healthy aging using open-ended questions. These culturally specific conceptions of health are considered as predisposing factors that shape participants' perceived health needs. Four subthemes include physical function, psychological well-being, social support, and cognitive function (Table 1). The cultural views of health may further affect healthy aging needs of Chinese older adults.

**(a) Physical Function**

When asked to describe characteristics of health, participants primarily mentioned aspects of physical mobility and being free of illnesses. They associated signs of aging with the inability to walk unaided. Study participants described the experience of aging as a gradual process of physical strength deterioration that often leads to feelings of discomfort. This was expressed mostly in terms of lack of energy, immobility, and chronic pain. To some, the realization of aging was unwelcomed. One participant portrayed his aging experience as such: “when a man grows old everything changes. Your movement slows down. Your energy diminishes… your mind is willing but your body fails to follow… everything deteriorates.” Additionally, Chinese older adults interpreted aging with reference to the Daoism philosophy of “emptiness”; that is, every person goes through the cycle of illness and pain. The key is to take it easy and accept their “fate.” As one participant commented, “(Aging) is like when the machine (my body) turns old, it cannot be successful.”

**(b) Psychological Well-being**

Study participants placed emphasis on the relationship between mental outlook and physical health, especially in terms of how a positive attitude contributes to healthy aging. One participant stated: “the most important treasure in a man's life is health… a healthy spirit can promote and lead to a good and healthy physique.” Despite the desirability of an upbeat mental outlook, many participants expressed concerns about psychological distress. They feared the feelings of “being bored” or “being left alone.” One participant, residing in the elderly apartment, described her observation on the community members' day-to-day lives: “i feel that the neighbors around us are lonely. I see they are bored. Their mobility is handicapped, and they appear like they are sitting inside a prison… I see that they are lonesome.”

**(c) Social Support**

When probed further into the reasons why participants may feel distressed at times, many of them reported that the dissatisfaction and loneliness stem from lack of social support. We found that family support was identified as the most desirable relationship that occurs through older adults' social network. One woman explained: “if your son and daughter-in-law are kind to you, you naturally feel happy. Even if you do not have money, you still feel happy. But if they are bad to you, you will not feel good even if you have money.” Participants also expressed feelings of social isolation within their own families because their adult children reside at a distance, and some believe it is a result of immigration, that “due to the influence in America, sons and daughters do not concern about parents.” Whereas participants were relatively vague relating the support they receive from family, friends, and neighbors, many were highly appreciative of community social service providers. Many participants commented that they would make a visit to the local community service center to seek assistance, or make friends with their peers through attending programs such as Tai Chi, dancing, and calligraphy lessons.

**(d) Cognitive Function**

Participants also expressed concern about losing the ability to remember things in their old age. Participants described the deterioration of brain in terms of forgetfulness and dementia. They believed that conforming to daily practices, such as keeping a healthy diet, exercising daily, and playing mahjong, would help maintain their being cognitively alert. Dementia and Alzheimer's disease often carry a social stigma among Asian cultures, leading families to shun formal diagnosis which increases the burden of both family and patients [45, 46]. Participants described the feeling of shame in terms of the relationship between dementia patients and their caregivers. One female participant stated: “I believe that in a non-Chinese family, members in the family would no doubt bring family members who suffer from dementia to see a doctor. But we Chinese love to save face… Chinese would not let other people know about the condition of the patients.”

### 3.3. Knowledge and Barriers to Health Care Services in the Community (Enabling Factors)

When asked about their experiences in clinical encounters in the United States, participants expressed many frustrating instances. The lack of access to health resources may further inhibit participants from performing behaviors to meet their health needs. Regarded as enabling factors, barriers of health services include cultural and linguistic obstacles, affordability, and availability of services (Table 2).

**(a) Cultural and Linguistic Barriers**

Accessing formal health care service for Chinese older adults is often impeded by language barriers. Lacking English proficiency to communicate with health professionals poses a serious hurdle for the majority of participants. Participants indicated that they need to depend on their caretakers, primarily their adult children, for translation and emotional support. Much to their regret, however, many participants reported that their health-care seeking behaviors were often not facilitated by family members primarily because of adult children's demanding workload or overlapping work schedules with clinic hours. Participants feared they may “increase the burden” or “bother” their children. A participant described his help-seeking behavior as follows: “If you need your sons to bring you to the doctor's clinic, he would have no time because he has to work. If you cannot find someone else, then you just have to wait until he comes home.” Participants indicated that the absence of adult children's assistance further limits their mobility. It also results in their being forced to seek formal social support assistance or to take public transportation in the city, which was troublesome.

**(b) Affordability of Services**

The inability to predict the costs of medical visits was troublesome for participants. Most participants in the sample were of low-income status and eligible for public insurance; however, many failed to understand these public benefits and indicate that the system was complicated and potentially expensive. The newly arrived participants, in particular, felt bitter about the services. As one participant commented, “We have nothing and yet we need to pay for the medication expenses.”

Utilization behaviors differed between the insured and uninsured older adults. Among those fully-covered participants, health insurance was utilized for major illness, rather than preventive or regular health care. Owing to the increased health problems in later life, some participants felt fortunate about having insurance coverage in America, stating that the US health care system benefits low-income older adults. One participant accounted for her US experience as follows: “my legs were broken twice in the US if that happened in China, my children and I would not be able to afford the surgeries. Here in the US I do not have to pay a single cent…”

**(c) Availability of Services**

Some participants were dissatisfied with US health care services due to the long wait and inflexibility of appointments. Particularly, the prolonged waiting period for an appointment often frustrated older adults and in some cases, it led to a worsening health condition. Sometimes they found it necessary to “predict” the timing that they might feel unwell, so that prompt treatment could be secured. A female participant commented, “They (US health care providers) are not letting you see the doctor today, unless you are going to the emergency room… then you surely need to go to the emergency room (because the situation worsens).”

Participants often sought quick results in America and found treatment unsatisfying. One participant reported the following: “like suffering from nerve pain… if you are in China, you just visit the clinic to get a pain killer shot. Over here the waiting period is not short. I went there and it took several hours. Going to the hospital for minor ailments is not convenient.”

### 3.4. Community's Perception on Health Sciences Research

In addition, we explored community's knowledge of health sciences research including participants' perception of research capacity building and attitudes regarding research recruitment and retention processes (Table 3). Understanding Chinese older adults' perceptions toward health sciences research helps shed light on whether community members support health improvement through biomedical research. Our analysis shows that community members have, in general, positive attitude toward health sciences research. Future recommendations in research endeavors were also discussed.

**(a) Positive Attitude**

Many participants expressed positive attitudes towards research. They were familiar with the potential benefits of health sciences research projects and there was a shared positive sentiment in the group towards research. One participant stated that: “we reflect our opinions so that the US government can have improvement… any social needs and problems can be improved upon and researched on… it is a benefit to everyone.”

Additionally, participants expressed their needs from a culturally specific vantage point, such as the demand to have more Mandarin- than Cantonese-speaking personnel which would better reflect the subgroup diversity. Participants stressed the needs of cultural relevance and cultural sensitivity of survey questions. For example, “If it is something to research on life saving methods for Chinese, then there is no fear to talk about it. Because the Western doctor himself has not experienced that type of illness, so he does not know what it is like. It is a good thing to talk to doctors about it and there should be no concern.”

**(b) Negative Perception and Future Recommendations**

Although most participants perceived research in a positive light, there remained a few concerns related to recruitment and intervention that may deter research participants from full involvement. Some negative impressions emerged based on the following reasoning: unclear research design and instructions, unknown or uninformed research results, and disclosure of privacy. As one participant stated, “you (researchers) need to provide the participants with instructions after the survey and let them know the outcome of the participation.”

Due to the low research participation of Chinese older adults, participants suggested a number of recruitment methods to improve results. Most participants agreed that recruitment through community service centers may be the most effective and efficient recruitment strategy. Advertising through community health forums and word of mouth of opinion leaders, through Chinese benevolent associations or community clinics, utilizing community news media, and expanding recruitment sites to elderly apartments may increase involvement. The key to ensure project success, as one older adult put it, is “to be relevant to the community's needs.”

## 4. Discussion

Successful healthy aging planning and initiatives must be based on a thorough needs assessment. In this first community-based participatory research (CBPR) study of health needs among Chinese older adults in Chicago, we identified priority community health concerns based on Precede-Proceed framework. The application of this framework was useful because our assessment findings were suggestive of the interplay of Chinese older adults' cultural perception on health and their behavior toward unmet health needs. Our findings show that Chinese older adults expressed their cultural conceptions of health in terms of physical function, psychological well-being, social support and cognitive function, which influences their perceived needs of health. The availability, affordability, and cultural barriers toward health care services were major negative enabling factors that inhibit Chinese older adults from fulfilling health needs. We also discussed the impact of health sciences research for designing culturally appropriate interventions toward the health needs of Chinese aging population.

## 5. Contribution to the Field

Based on qualitative analysis, this investigation provides unique insights on the health needs of a fast-growing older immigrant group in the United States. First, our study expands the prior findings in the understudied cultural variation of health and aging. Our results reveal that Chinese older adults tend to have minimal anticipation in aging successfully, which may influence their attitudes toward health behavior and their use of health care. Consistent with prior research, Chinese older adults in this study use a holistic approach to conceptualize health. They perceive the balanced interconnection between mental outlook and physical health as a successful model for aging [47, 48]. The Ying and Yang equilibrium in terms of harmony of body, mind, and soul was perceived as a crucial element for well-being [11, 21]. However, it is noteworthy that their notion of health was often accompanied by a sense of helplessness and a feeling of no control over the course of life and illnesses. The insecurity about physical function decline and cognitive impairment may be associated with placing less importance on health-seeking behaviors [49]. 

Moreover, our analysis shows that Chinese older adults perceive social support as a major unmet health need, along with physical and cognitive functions. Although filial obligation is a highly valuable belief within the context of Chinese culture, for Chinese older adults in our study, the traditional pivotal supporting role of family, especially adult children, seems to be changing [50]. Whereas prior research suggests family and kin as the chief social support resources among Chinese older adults [51], our findings suggest that the main resource of social support among this group of older adults has shifted toward formal social support mechanisms provided by the community service center. Due to the discrepancies between changing cultural demands and the significance of traditional values, older US Chinese immigrants in our studies may be pushed to alter their expectations of filial care in the contemporary American culture, which in turn could negatively impact the health and well-being of older adults. This cultural disconnection between older adults' high expectation of family support and the actual amount of care and support they receive from family members may trigger family conflict and increase the risks of emotional distress among Chinese older adults. Furthermore, the lack of familial support and attendance further deepen their barriers to health care services. 

Our findings also suggest that barriers including language and communication difficulties, issues of transportation, and lack of familiarity with the US health care system are negative enabling factors that inhibit participants from actively seeking health care services. Whereas the average number of years residing in the US among study participants was twenty, we speculate that the responses of older adults in this study reflect less assimilation than would have been anticipated or reported in other studies [13]. This phenomenon may reveal that despite the growing numbers of Chinese older adults whose cultural values toward aging and health-related practices are shifting toward the US mainstream, there are still groups of Chinese older adults without adequate skills or resources to perform health behaviors toward their needs. The identification of the barriers to the use of services as perceived by older adults is a first step toward planning of appropriate services.

Last, the ability to reach and investigate the health needs of this vulnerable population was facilitated through the use of CBPR. To our knowledge, this health need assessment is the first reported CBPR study with Chinese older adults. Conducting a participatory approach allows us to place this sensitive cultural context as central in the process of scientific inquiries, including the planning of research, collection of data, and analysis and interpretation of data [52, 53]. From conducting partnership meetings, focus group discussions, to organizing health educational outreach programs to disseminate our findings, community members were able to exchange and identify ideas of community health needs in settings that are comfortable and familiar for Chinese older adults. Hence, our project has gained trust among the community which is a necessary step in research with this community. The synergistic collaboration, in turn, offers an opportunity for researchers to hear from Chinese older adults on the meanings and the needs of healthy aging that truly reflect the community's voice.

## 6. Limitations

There are several limitations to this study. First, the data used in this investigation are from a small sample of Chinese older adults. A future larger population-based study is needed to confirm our findings. Second, the sample used was purposively selected from Chicago's Chinatown community, and hence, our results may not be generalizable to other Chinese populations, including suburban groups, Chinese ethnic minority groups, or rural Chinese populations, as they may be subjected to varying degrees of social and economic influence [54]. Research suggests that the diversity among overseas Chinese community is vast [20]. US Chinese communities are predominantly composed of foreign-born Chinese persons and comprises at least five generations, of varying acculturation degrees, settling in from mainland China, Taiwan, Hong Kong, Vietnam as well as refugees of Chinese descent in Southeast Asia, Latin America, and Caribbean countries. These sociodemographic characteristics further call for culturally sensitive measures for researchers concerned with promoting healthy aging in Chinese community [1, 22, 41, 42]. 

Third, we did not collect any quantitative information of the health status, nor utilization of health care services which may have been useful in improving our current understanding of Chinese older adults' health-related behavior. Further mixed methods studies are needed to combine the rigor of both methodologies to produce multiple viewpoints and perspectives. Fourth, we did not have information on the perception and expectations of changing cultural values in US Chinese older adult. In particular, future studies are needed to explore filial piety, the core of Chinese culture, not only among adult children's observance of these traditions, but also the potential changing filial expectation of older adults, with respect to important health outcomes. Nonetheless, this qualitative study generated useful insights about the aging experience and cultural perceptions among older adults that lay the groundwork for future research on the well-being of the Chinese aging population.

## 7. Implications

Our study has several important health service implications. First, cultural relevancy of health interventions is important in the context of Chinese community. Cultural sensitivity in health intervention programs embodies more than matching languages and locations preferred by a targeted community. Rather, incorporating the cultural, social, and environmental forces that affect health behavior in the targeted community contributes to the salience of policy impact [55–57]. In the case of Chinese community, an effective program will need to provide culturally sensitive model of successful aging tailored toward Chinese older adults, in order to counteract negative beliefs such as fatalism or the notion of “emptiness” which is ingrained in Chinese culture. Respecting older adults' cultural values on health may help lower their cultural barriers in accessing health care services [20]. 

Second, it is imperative to increase Chinese older adults' resources in health services. Our findings suggest that community service centers may play a crucial role in empowering Chinese older adults. For older adults with limited understanding toward US health system, community service centers that provide bicultural and bilingual services are needed to encourage older adults to take proper action toward their own health needs. Furthermore, social workers are well-positioned to assist Chinese older adults who are in need of support network. Social service agencies could facilitate the ability of family members of Chinese older adults to offer adequate support by providing intergenerational services. Community programs that help in nurturing familial obligations as well as community caring values may help to bridge the gaps.

Third, we suggest that it is imperative to improve the cultural understandings between health providers and minority older adults. At present, medical education has developed cultural competence training to prepare future health professionals. We suggest that such curriculum should also encourage health professionals to become better listeners and students of the patients. The ability to listen to the older adults is essential to comprehend the cultural variations of health and aging in which minority older adults are coming from [58, 59]. For instance, our analysis show that older adults often compare their medical visits experience in the US to that in China, where quick results and improvements are anticipated. Furthermore, health concerns among Chinese older adults are often intertwined with their unique Confucianism and Daoism cultural beliefs. In situations as such, we suggest that health professionals' humble and compassionate clinical approach is critical in terms of forming a therapeutic alliance [60]. The result will benefit Chinese older adults' understanding toward Western treatment and compliance of medicine that truly address their unmet health needs.

Last, despite a wide recognition that rigorous research with Chinese older adults may be difficult, our study identifies important factors that will contribute to improved involvement in further biomedical and behavioral research. Chinese older adults are aware of the benefits of health sciences research, given that the conduct and content of research is culturally sensitive. The unique language, culture, and familial qualities of this group warrant further differentiation of their needs.

## 8. Conclusions

Translation of research knowledge into locally relevant policy and action is the primary strength as well as the ultimate goal of a CBPR study. In summary, our qualitative study on health needs contributes to the emerging literature of the complex nature of aging among immigrant older adults and carries important policy implications. We believe this health needs assessment through a CBPR model is applicable to other communities and aging groups.

## Figures and Tables

**Table 1:** Community's cultural views of health.

Themes	Subthemes	Representative statements
Physical function	Body is like a machine; its deterioration is unstoppable and irreversible	“When a man grows old everything changes. Your movement slows down. Your energy diminishes. The heavy loads you lift up at youth will overwhelm you at your old age. Your mind is willing but your body fails to follow.” “If the feet of the elderly have no strength, then they will fall easily. That is why we say the feet get old faster than the person.”
	The realization of aging is not welcomed, and some tended to be fatalistic about physical deterioration	“When the machine turns old, it cannot be successful.” “A person's life is full of ups and downs and contrary to your expectation of being cordial and friendly, it often turns sour and empty. Therefore, we need to have an empty philosophy of life and accept our fate.”

Psychological well-being	High spirits help promote a healthy body. Body, soul, and mind are inseparable entities	“Our life goes through birth, old age, illness, and death. The key is whether we are happy or not.” “The most important treasure in a man's life is health. A healthy spirit can promote and lead to a good and healthy physique.”
	The experience of loneliness and boredom tend to be common, and may lead to distressed situations	“I feel that the neighbors around us are lonely. I see they are bored. Their mobility is handicapped, and they appear like they are sitting inside a prison.” “I feel the most terrible thing for old people is to be left alone.”

Social support	Family support is most desirable and valued. However, it is not always available. Participants are ill-prepared to adjust to the cultural and generational changes compounded by immigration impact	“If your son and daughter-in-law are kind to you, you naturally feel happy. Even if you do not have money, you still feel happy. But if they are bad to you, you will not feel good even if you have money. Even if you live in the elderly apartments, you would go downstairs to stir your complaints, and you would still feel bad.” “My understanding is that for most people, their sons and daughters do not concern about their parents. That is due to the influence in America. It is to take care of yourself only.”
	Community service center becomes the resource for support	“Introduce them (bored older adults) to join activity programs (in the community service center). Whatever their preference, let them join that type of activities such as exercise Taichi, singing, dancing, painting, and many other programs. Or let them vent and voice their complaints. It helps to have an outlet.”

Cognitive function	Impaired cognitive function is perceived as a health concern, in which dementia is most severe and brings shame and burden to the family	“Sometimes I keep looking for something for an hour without success. When I finally find it, it is right under my nose. I put it in my pocket. A moment later, I look for it again. This happens again and again.” “I believe that in a non-Chinese family, members in the family would no doubt bring family members who suffer from dementia to see a doctor. But we Chinese who love to save face, always try to solve the problems at home. Chinese would not let other people know about the condition of the patients, such as uncontrollable urination and bowel movement.” “You might not agree with me. But I would rather swallow a pill and die as long as it is not too painful. Everybody has to die. Why bother people too much? Swallow a pill and pass away in your sleep.”
	Although cognitive impairment may come with age, practices could prevent impairment by conforming to exercises and practices	“I realize that as we approach old age, degenerating is unavoidable. But you can do something to slow down the process. Force yourself to exercise your brain to keep your mind sharp.” “I think exercise is very important. Exercise should be a part of our daily lives. Use your head to think. Use your imagination. Read more books. Read newspapers. Play mahjong.”

Note: table presents a summary of findings from focus group discussions among participants.

**Table 2:** Knowledge and Barriers to Health Care Services in the Community.

Themes	Subthemes	Representative statements
Cultural and linguistic barriers	Major barriers in terms of language and culture result in older adults' dependency on others when it comes to medical visits	“The biggest problem is the language barrier. We feel helpless since we do not speak English, and we cannot resolve matters without English. Even if you understand English you would not comprehend their technical terms.” “The people I contact are few. I don't want to bother my children.”
	Immediate family support may not always be available to assist older adults with medical visits	“If you need your son to bring you to the doctor's clinic, he would have no time because he has to work. If you cannot find someone else, then you just have to wait until he comes home.” “To ride the bus you need to know how to get there… you need to draw it on a piece of paper with the directions. I was to take bus no. 4, and it would transfer three times. But still I did not make it (to the hospital). I was lucky to return home from the final bus stop.”

Affordability of services	The health care system is poorly understood. The utilization and degree of satisfaction differ depending on older adults' insurance coverage and length of residence	“As new comers, we have nothing. My mother-in-law is old, and, moreover, I am old too, I am sixty eight years old. As we age, the most critical thing is medical treatment and benefits. Getting your white card, it would not be a big deal even if you are sick. We depend on the medical supply by mail. It is tough to see a doctor. Besides it is far away. And not knowing English, it is difficult to visit a doctor. The medical expenses are high.” “It's like we have to pay for everything. We have nothing and yet we need to pay for the medication expenses.”
	For those who are fully insured, they feel fortunate compared to that of their counterparts in China	“Buying insurance is now only getting started in China. Previously, I bought insurance. Now I apply for low-income status. So I do not have to buy insurance and it is so much better. America is definitely taking that route. You have no money. The government helps out with a low income status. If you apply, it will not cost you any money.” “I think the most pleasant thing for me in the US is what happened to my two legs. My legs were broken twice in the US if that happened in China, my children and I would not be able to afford the surgeries. Here in the US, I do not have to pay a single cent. They even provided me the walking equipment and the toilet pans. I think this is the best thing happened to me. I feel very happy and thankful for that.”

Availability of services	The long wait and inflexibility of health care services often frustrated participants	“I think the waiting period to see a doctor is too long. It takes several hours and you are not seeing your doctor right away. Definitely it is too long.” “I think there is something that America is not comparable to China. That is the appointment system. They are not letting you see the doctor today, unless you are going to the emergency room! They will not let you see the doctor right away; it would take a week. Then you need to go to the emergency room. I think that situation is not good.”
	The expectation toward medical visits is often compared to older participants' experience in China where quick improvement is anticipated	“In Shanghai, if I do not feel good, I can go to see the doctor right away. Over here, this appointment thing, you would need two weeks to see your family physician. He has his vacations and you need to wait two weeks. But hey, I am sick… I have this illness and I need to see the doc right away. I think over here, you need to go to the hospital, the UIC Hospital. It is because of the insurance that requires you to visit the hospital. Today you can visit this hospital and tomorrow you can visit that hospital. And that is OK.” “Like suffering from nerve pain… if you are in China, you just visit the clinic to get a pain killer shot. Over here the waiting period is not fast. I went there and took several hours. Going to the hospital for minor ailments is not convenient.”.

Note: table presents a summary of findings from focus group discussions among participants

**Table 3:** Community's Perception on Health Sciences Research.

Themes	Subthemes	Representative statements
Positive attitude	Participants in general commented that research could bring potential benefits to the community at large	“We reflect our opinions so that the government can have improvement. So there is no harm in joining the survey. Any social needs and problems can be improved upon. If you have a concern, just express it so that the government can get better. It is a benefit to everyone.”
	There exist needs to explore culturally specific research tailored toward understudied ethnic minority population such as Chinese	“If it is something to research on life saving methods, then there is no fear to talk about it. Because the Western doctor himself has not experienced that type of illness. So the doctor does not know what it is like. This is a good thing to talk about it and there should be no concern.”

Negative perception and future recommendation	Concerns toward research include not knowing the relevant outcomes and benefits and fear of disclosing privacy	“You need to provide the participants with instructions after the survey and let them know the benefits of the participation in understanding the signs and symptoms of the disease. With clear directions, the number of participants will increase.” “Yet people's concern is that after the survey, their privacy will be taken away.”
	It is suggested that through word of mouth and health forums it will be best to involve the community in research	“We can advertise in the newspapers. We can request people to offer their opinions. Then we can collect their comments and information.” “This ‘word of mouth' type of advertisement can be spread through the community organizations.” “Promote more activities. Let people have more contacts. Now there are more Mandarin speaking people from the Mainland. Previously there were few.” “In terms of language, more people speak Mandarin. It would be great like you who can speak in both dialects.” “To do that we have to be relevant to the community's needs. Otherwise it would turn into hearsays and chatters.”

Note: Table presents a summary of findings from focus group discussions among participants.
